# Major infections in children with systemic lupus erythematosus

**DOI:** 10.1186/1546-0096-10-S1-A20

**Published:** 2012-07-13

**Authors:** Ana Patricia Costa Reis, Simona Nativ, Josephine Isgro, Lisa F Imundo, Lisa Saiman, Andrew H Eichenfield

**Affiliations:** 1 Columbia University Medical Center, West Orange, NJ, USA; 2 Columbia University Medical Center, New York, NY, USA; 3 Santa Maria Hospital, Lisbon, Portugal

## Purpose

Careful monitoring for infection is warranted in children with SLE, especially those receiving immunosuppressive drugs. Infection is not only the leading cause of death in pediatric SLE, but it is also associated with morbidity. It has been observed that recurrent major infections can predict poorer disease outcome. Prevention of infection and consequent disease damage is therefore a major challenge in managing SLE. The primary goal of our study is to describe the pattern of major infections in a cohort of patients with pediatric SLE. Our secondary aim is to identify whether there is an association between major infections, immunosuppressive treatment, and disease damage.

## Methods

Retrospective chart review of the clinical characteristics, disease activity (SLEDAI), major infections and disease damage index in 120 SLE patients diagnosed before the age of 18 and followed by our division during the years 2007-2009. The clinical course and treatment for the six months prior to and after each major infectious episode were analyzed to define any lupus disease flare and its management. A major infection was defined as one that required treatment with parenteral antimicrobial agents or a prolonged course of more than one week. Disease damage was evaluated by the SLICC/ACR Damage Index (SDI). Chi-square test and unpaired t-test were used for categorical and continuous data, respectively (PASWStatistic18.0).

## Results

The majority of the children (53%) were diagnosed when they were between the ages of 10-15 years. The female to male ratio was 3.4:1. There was visceral involvement in 60% (72): 49% (59) had renal involvement, 12% (15) had neurologic disease, and 5.8% (7) had a history of stroke. Organ damage (SDI >1) occurred in 32.5% (39). There were 101 major infections affecting 44 patients (37%), the most common being skin and soft tissue infections (18), pneumonias (17), urinary tract infections with fever (13), herpes zoster (8) and sepsis (7). There were no infections with Pneumocystis jiroveci. One patient died of disseminated CMV infection. SLEDAI at diagnosis, cumulative dose of prednisone, prior treatment with cyclophosphamide or mycophenolate mofetil were each associated with the occurrence of major infections (P <0.005). There was no association between treatment with azathioprine and major infection. An association between major infection and organ damage was also found (P <0.005).

## Conclusion

In a large cohort of children with SLE, major infections were found to be common, associated with active disease and its treatment, and resulted in noteworthy morbidity and mortality.

## Disclosure

Ana Patricia Costa Reis: None; Simona Nativ: None; Josephine Isgro: None; Lisa F. Imundo: None; Lisa Saiman: None; Andrew H. Eichenfield: None.

